# Development of the Web-Based Spanish Version of the Barthel Index in Patients with Multiple Sclerosis

**DOI:** 10.3390/ijerph192113965

**Published:** 2022-10-27

**Authors:** Sandra Aguilar-Zafra, Tamara del Corral, Juan Antonio Valera-Calero, Patricia Martín-Casas, Gustavo Plaza-Manzano, Ibai López-de-Uralde-Villanueva

**Affiliations:** 1 Departamento de Fisioterapia, Facultad de Ciencias de la Salud, Centro Superior de Estudios Universitarios La Salle, Universidad Autónoma de Madrid, 28049 Madrid, Spain; 2 Motion in Brains Research Group, Centro Superior de Estudios Universitarios La Salle, Universidad Autónoma de Madrid, 28049 Madrid, Spain; 3 Téxum S.L Physiotherapy Center, 28821 Madrid, Spain; 4 Department of Radiology, Rehabilitation and Physiotherapy, Faculty of Nursing, Physiotherapy and Podiatry, Universidad Complutense de Madrid, 28040 Madrid, Spain; 5 Instituto de Investigación Sanitaria del Hospital Clínico San Carlos (IdISSC), 28040 Madrid, Spain; 6 VALTRADOFI Research Group, Department of Physical Therapy, Universidad Camilo José Cela, 28692 Villanueva de la Cañada, Spain

**Keywords:** disability evaluation, activities of daily living, multiple sclerosis, web-based intervention, test-retest reliability

## Abstract

Background: The aims of this study were to develop a web-based Spanish form of the Barthel index (BI), to evaluate its psychometric properties and stability over time (test-retest), and to determine minimal detectable change (MDC) in patients with multiple sclerosis (MS). Methods: Participants answered the BI on two forms (web-based and face-to-face interview), 7–10 days apart. The internal consistency was evaluated using Cronbach’s alpha, and intraclass correlation (ICC) and kappa (κ) coefficients were used to investigate the agreement between both forms. Results: 143 participants were included. The Spanish web-based form of the BI showed excellent agreement between both forms for each item (κ = 0.86 (0.79 to 0.92), and for total score (κ = 0.87 (0.81 to 0.93); ICC = 0.99 (0.98 to 0.99). The internal consistency was good–excellent (Cronbach’s alpha = 0.89 (0.86–0.91)). The stability over time was adequate, the agreement of each item was κ = 0.63 (0.52–0.74)), and for total score (ICC = 0.97), determining a MDC_95_ of 12.09 points. Conclusions: The Spanish web-based form of the BI is a valid and reliable tool to assess functionality and can be applied in both formats in patients with MS. A total score difference of more than 12 points was found to indicate a deterioration or improvement in the patient’s functionality.

## 1. Introduction

Multiple sclerosis (MS) is an inflammatory disorder characterized by demyelination of the central nervous system, which causes sensory, physical, and psychological damage, as well as impairment of functional capacities [1]. It is the most common non-traumatic disabling neurological disease in young adults, especially in women [2]. MS is associated with a substantial negative impact on quality of life and activities of daily living [3], leading to varying degrees of disability and loss of functional independence over time [4]. Both the initial disease course and the disability-related factors have been found to be predictors of the malignant course of the disease [5], which have a major impact on healthcare systems, with more substantial economic consequences than treatment [6]. 

Many existing scales quantify the patient’s degree of disability and measure their dependence upon others in basic daily activities. The Expanded Disability Status scale (EDSS) [7] is the most widely used tool for the measurement of disability and impairment caused by MS. However, it might not adequately reflect the patient’s own perceptions of the impact of symptoms on their overall health status and their quality of life, given that some functional areas are not sufficiently assessed, such as mood, energy levels and quality of life [8]. The Incapacity Status scale [9] is another method of quantifying disability by testing the ability to perform activities of daily living; the functional independence measure [10] records the severity of disability. However, none of these scales are sensitive to the types of changes that occur in MS [11]. 

Based on these problems, the Barthel index (BI) can be considered a solution. The BI is a 10-item activities of daily living ordinal scale used primarily for disability measurement [12]. The values assigned to each item are based on the time and amount of physical assistance required for a patient to perform a common daily activity. It is highly recommended because is easy to perform, fully self-managed, and can be completed by caregivers, thus increasing user compliance; it is not dependent on an updated report from a neurologist [13]. The BI is highly correlated with the EDSS, but it is more sensitive in detecting changes [13,14]. The BI is also strongly associated with in-hospital mortality, discharge destination, and length of stay [15], and it has proven its prognostic utility [16].

The BI has been shown to be a valid tool for assessing the degree of functional disability in patients with MS [13,17], as well as in patients with other neurological pathologies [18,19,20], when applied in a face-to-face interview format. However, the COVID-19 pandemic has forced all healthcare providers to be adaptive and innovative in their patient care so as to maintain adequate social distancing and to reduce the risk of disease transmission, especially in vulnerable populations such as patients with MS. Among the measures taken, hospital visits have been limited to a minimum, and telematic consultations have increased [21], which emphasizes the need for non-traditional self-administered pencil and paper format assessment tools. There are validated versions of the BI adaptations for application to telephone and postal formats [22,23,24]. However, a web-based form of the BI could be a suitable alternative for the evaluation of patients with MS who have restricted mobility because it would consume fewer human and material resources. Currently, the Spanish version of the BI has only been validated in elderly people by means of a combined application in the face-to-face interview and postal formats [25], It is important to note that the validity of a scale is not a property of the tool itself, but of the interpretation of the assessment instrument with a specific population [26]. Thus, we considered it relevant to develop and evaluate the metric properties of a Spanish web-based form of the BI in patients with MS.

The aim of this study was to develop a web-based Spanish form of the BI and to evaluate its psychometric properties in patients with MS. An additional aim was to evaluate the stability of the scale results over time (test-retest), as well as to determine the minimum difference necessary to consider that there has been a real change (minimal detectable change, MDC).

## 2. Materials and Methods

### 2.1. Study Design, Setting and Participants

This was an observational and longitudinal study in accordance with the criteria established by the Consensus-based Standards for the Selection of Health Status Measurement Instruments guidelines [27]. The study was approved by the ethics committee of the Complutense University of Madrid (registration number: CE_20220120-09_SAL) and was conducted in accordance with the Declaration of Helsinki. 

The sample was recruited from the following centers in the Community of Madrid area: Móstoles Multiple Sclerosis Association, Téxum S.L Physiotherapy Centre, Parla Association of Multiple Sclerosis, Collado Villalba Association of Multiple Sclerosis and Multiple Sclerosis Foundation, using a method of consecutive sampling by convenience. The selection criteria established were as follows: clinically definite MS according to 2017 Thompson criteria [28]; at least 18 years of age; clinical stability (no relapses within 30 days prior to the inclusion in the study); ability to speak and read the Spanish language; ability to understand the questionnaires and scales; and having internet access. Written informed consent was obtained from all the participants.

### 2.2. Study Procedures

An independent investigator, by means of a telephone call, ensured that the patients met the inclusion criteria for the study and informed them whether they should complete the web-based form of the BI before or after attending the face-to-face interview. This assignment order was generated randomly prior to the interview. During this phone call, the investigator explained the study procedure, asked the participant to continue with their daily routine, and requested an e-mail address to send them reminders and instructions for the correct execution of the study.

During the face-to-face interview, an evaluator administered the BI, the EDSS and the EuroQol-5D questionnaire (EQ-5D-5L), following a randomized order. If the patients were physically unable to respond, their caregivers did so. The examiner recorded the patient’s anthropometric and sociodemographic characteristics, as well as specific clinical characteristics (e.g., type of MS, comorbidities). For the online completion of the web-based form of the BI, an e-mail was sent to the patients that included their identification number along with a direct link to the web page where they had access to the web version of the scale. This email was sent 3–4 h before or after their face-to-face interview (during which time they were encouraged to engage in a quiet task such as reading or watching TV to prevent discussion of either scale). For the cases in which they had to complete the BI beforehand, it was a prerequisite to confirm that they had done so before proceeding to the interview. Similarly, patients who had to complete the web-based form after the interview were reminded that they would receive an e-mail after 3–4 h, in which they would have access to the scale. Finally, after 7 days, the patients were sent another reminder e-mail asking them to complete the web-based form of the scale within the next 3 days.

### 2.3. Outcomes

#### 2.3.1. Barthel Index 

The BI is an ordinal scale comprising 10 activities of daily living (feeding, grooming, bathing, dressing, bowel and bladder care, toilet use, ambulation, transfers, and stair climbing) [12]. It was scored in steps of 5 points, for a maximum total score of 100, indicating that an individual is fully independent in physical functioning; the lowest score is 0, indicating an individual who is totally dependent for performing normal physical activities. The Spanish version of the BI presents adequate psychometric properties (Cronbach’s alpha ≥ 0.88) [25].

#### 2.3.2. Expanded Disability Status Scale 

The EDSS is a 20-step ordinal scale, grading disease severity from 0 (normal status) to 10 (death due to MS) [7]. This rating is based on the medical history and the neurological examination. The EDSS score was classified as follows: mild disability (EDSS score = 0–3.5), moderate disability (EDSS score = 4–6.5) and severe disability (EDSS score ≥ 7). Evidence supports the validity of the Spanish version of the EDSS [29]. 

#### 2.3.3. Health-Related Quality of Life

To assess the patient’s quality of life, we employed the EQ-5D-5L questionnaire [30], which consists of 5 dimensions (1, mobility; 2, self-care; 3, usual activities; 4, pain/discomfort; 5, anxiety/depression), with 5 response options based on severity level, ranging from 1 to 5. Based on these 5-dimension codes, an index score is provided, ranging from 0 (death) to 1 (full health). The patients also rated their current overall health on a visual analogue scale, ranging from 0 (poorest imaginable health) to 100 (best imaginable health).

### 2.4. Sample Size

According to the criteria of several authors, a sample size of at least 100 patients would be sufficient to determine the validity of the Spanish web-based form of the BI, given that this scale is composed of 1 factor made up of 10 items [31,32,33]. Other authors recommend having at least 10–15 cases per observed variable to achieve an adequate sample size [34]. Hence, a sample size of 100 patients would be sufficient to satisfy both criteria. However, we decided to reach a sample size closer to 150 patients to increase statistical power. Thus, we ultimately decided on a sample size of at least 130 patients to obtain more stable results, being less likely to be affected by outliers.

### 2.5. Linguistic Adaptation

The linguistic adaptation of the web-based form of the BI was performed according to the usual recommendations for translating and culturally adapting questionnaires based on patient-reported measures [35].

Initially, two native Spanish speakers independently translated the paper-based form of the BI into Spanish. A prerequisite was that the original meaning of each item was preserved, as well as the use of language that facilitated understanding. A multidisciplinary panel of neurorehabilitation experts evaluated, ratified, and merged the two Spanish translations of the scale. Subsequently, two native bilingual English speakers back translated the Spanish web version of the BI into English. Both back translations were compared with the original by the committee of experts who ensured that semantic equivalence was maintained.

Lastly, a pilot study with 40 patients with MS evaluated the clarity and feasibility of using the Spanish web-based form of the BI. Table S1 presents the Spanish web-based form of the BI.

### 2.6. Data Analysis

The data were analyzed using SPSS v. 25 software (SPSS Inc., Chicago, IL, USA). The level of significance was set at *p* < 0.05. 

#### 2.6.1. Validity

The internal consistency of the BI web-based form was evaluated using Cronbach’s alpha, with a value >0.7 being considered acceptable [36]. Percent agreement and kappa coefficients [37] were used to estimate concordance between the web-based form and the face-to-face interview (criterion validity) for each item score and for the categorical ranking of the total BI score. Kappa or weighted kappa was used for binary or multiple category scales, respectively. Kappa values were interpreted according to the Landis and Koch criteria [38]: poor agreement (<0.2), fair agreement (0.21–0.40), moderate agreement (0.41–0.60), substantial agreement (0.61–0.80) and almost perfect agreement (>0.80). Regarding the absolute value of the BI total score, the agreement between the web-based form and the face-to-face interview was examined using the intraclass correlation coefficient (ICC): excellent (ICC ≥ 0.90); good (0.90 > ICC ≥ 0.70); fair (0.70 > ICC ≥ 0.40); and poor (ICC < 0.40) [39]. 

Construct validity was assessed by analyzing the correlations between the total score of the web-based form of the BI with patients’ health-related quality of life (EQ-5D-5L), as well as with their disability status (EDSS), age and clinical characteristics. Spearman’s rho correlation coefficient was used to determine correlation: low correlation (rho < 0.30), moderate correlation (rho = 0.30–0.60) and strong correlation (rho > 0.60) [40]. The discriminant ability of the web-based form was assessed by comparing the scores obtained for disability status and health-related quality of life between the various grades of disability according to the BI, as well as by comparison of the total scale score according to sex and number of comorbidities. The group factor was analyzed using the Kruskal–Wallis test, and multiple comparisons were performed with the Mann–Whitney U test.

#### 2.6.2. Reliability

The test-retest reliability (7–10 days) of the web-based form of the BI was assessed as follows: (1) for the score of each item and the categorical score of the total scale, the percentage of agreement and kappa coefficients (kappa or weighted kappa) were used; (2) for the absolute value of the total scale score, the ICC was used. The standard error of measurement (SEM) and the MDC as 95% of the confidence interval (MDC_95_) were calculated following the same criteria as a previous study [41]. 

## 3. Results

The total sample consisted of 143 patients with MS (84 women and 59 men), of whom 74 had relapsing-remitting MS, 33 had primary progressive MS, and 36 had secondary progressive MS. Most patients reported a moderate level of disability as well as the presence of at least one comorbidity. Table 1 shows the sociodemographic and clinical characteristics of the patients included in the study. A total of 22 patients did not complete the web-based form of the BI within 7–10 days of their first assessment. Thus, the test-retest reliability of the online version was examined in a total sample of 121 patients.

### 3.1. Criterion Validity of the Web-Based Form of the Barthel Index 

The scores obtained in the web-based form of the BI showed almost perfect agreement with those obtained when the scale was administered by a face-to-face interview (Table 2). Specifically, the percentage of agreement between both forms for each item was ≥88%, obtaining a kappa coefficient of 0.86 (0.79–0.92). Regarding the agreement between both forms for the total score, a kappa coefficient of 0.87 (0.81–0.93) and an ICC of 0.99 (0.98–0.99) were obtained when this score was respectively established categorically and absolutely. In addition, the internal consistency of the web-based form of the BI was good-excellent (Cronbach’s alpha = 0.89 (0.86–0.91)). Therefore, the web-based form of the BI apparently presents adequate criterion validity.

### 3.2. Construct Validity

The convergent validity of the web-based form of the BI was adequate, given that the correlational analysis showed a statistically significant relationship between the total BI score and patients’ health-related quality of life (EQ-5D-5L), as well as with their disability status (EDSS), age, and clinical characteristics (type of MS, urinary disturbance, and number of comorbidities; see Table 3). All correlations between the BI were of moderate–large magnitude, with the exception of those observed with the patients’ comorbidities, which were low (rho < 0.30). The BI score showed a particularly high correlation with disability status (EDSS; rho = 0.769).

The Kruskal–Wallis test showed statistically significant differences in the web-based form of the BI score for the number of comorbidities (*p* = 0.016), disability status (*p* < 0.001) and health-related quality of life (*p* < 0.001) of the patients with MS. Specifically, patients with two or more comorbidities scored lower compared with those without comorbidities. Likewise, there were differences in the total BI score between patients with different degrees of disability status according to the EDSS, with patients showing lower BI scores as their degree of disability in the EDSS increased. In terms of the categorical BI total score, patients classified as ‘severely disabled’ or ‘very severely disabled’ showed statistically significant deterioration in their disability status and in their health-related quality of life than those classified with lower levels of disability. Similarly, patients classified as ‘moderately disabled’ had significantly poorer health-related quality of life and disability status according to the EDSS than those classified as ‘physically independent’ (*p* < 0.05). Multiple comparisons are presented in Table 4. 

### 3.3. Test-Retest Reliability of the Web-Based Form of the Barthel Index

The stability of the score obtained again in the web-based form of the BI at 7 days (test-retest) was almost perfect (Table 5), both when considering the score of each item separately (percent agreement ≥92%; kappa = 0.63 (0.52–0.74)) and when considering the categorical ranking of the total score (percent agreement = 86%; kappa = 0.63 (0.52–0.74)). In addition, with respect to absolute total score agreement, excellent agreement (ICC = 0.97 (0.96–0.98)) was found, determining a SEM of 4.36 points and a MDC_95_ of 12.09 points (relative MDC_95_ = 16%) for the total score of this online version.

## 4. Discussion

The present study is the first to assess the validity and reliability of the Spanish web-based form of the BI in patients with MS. The web-based items of the BI showed excellent agreement with each item in the face-to-face interview. The web-based form also showed excellent internal consistency, as well as adequate construct validity, given that its score was related to scales measuring related constructs and discriminated patients with various degrees of functional disability. The stability over time of the web-based form was adequate, this being the first study to report MDC in patients with MS. Thus, the web-based form of the Spanish version of the BI appears to be a valid and useful tool in clinical practice to determine the degree of functional disability in Spanish-speaking patients with MS and could be an alternative to the application of the BI in a face-to-face interview format.

The percentage of agreement for the Spanish version of the BI rating between the web-based and face-to-face interview forms was high (≥88%), with kappa values ranging from 0.86 in stairs to 0.99 in transfer. Prior studies have addressed the reliability of remote BI assessment in patients after stroke or other orthopedic conditions, most using the telephone interview [22,23,42,43,44,45], and two using the postal self-report version [23,24]. Supporting our results, most of these studies had a good agreement between conventional and remote BI scores [22,24,42,43,44,45]. A meta-analysis concluded that there was extensive evidence to show that paper and computer versions of self-reported questionnaires are equivalent [46]. The analyses of individual item scores indicate the lowest agreement on the item ‘climbing the stairs’, as in the Yeo et al. [44] study comparing the postal and conventional form in older adults, which could be attributed to differences in the meaning of the ‘flight of stairs’ item. Homes are often adapted to the needs of disabled individuals, eliminating obstacles or flights of stairs. Thus, the term ‘flight of stairs’ refers to a simple hand ladder, and its use is fairly difficult in patients with MS. 

Although no previous report on the web-based form of the BI was found, the internal consistency of this version is in line with other studies that evaluated remote versions of the scale by telephone interview [47,48]. It was also similar to that obtained for the conventional form of the BI by face-to-face interview in patients with MS [17] (Cronbach’s alpha = 0.89 vs. 0.86). This finding could be explained by the high agreement found between the face-to-face interview and the web-based form. However, the internal consistency was higher than the validated conventional Spanish version [25] performed in four different cohorts of older adults, which could be because the application of a scale in different populations can cause variations in its internal consistency [49].

The construct validity of the Spanish version of the web-based form of the BI was adequate. The scale showed good correlations with patients’ health-related quality of life, disability, and clinical features. In addition, the scale discriminated between patients with various degrees of disability and quality of life, demonstrating its discriminant validity. Our study confirms the good correlation observed previously between the EDSS and the BI [13,14] and the pattern of association with patients’ health-related quality of life, age and clinical features. Of note, the highest correlation was observed with disability status (EDSS; rho = 0.769). These results could be explained by the fact that the EDSS evaluates the disability status predominantly through a set of questions regarding walking ability and functional systems, and it has similar items focusing on some mobility aspects originating from the BI. In terms of the patients’ health-related quality of life, age, and clinical characteristics (e.g., type of MS and number of comorbidities), the correlations were moderate to large in magnitude. This strong correlation was expected because the ‘usual activities’ domain of the EQ-5D-5L questionnaire evaluates the same dimensions of the BI, including the most popular activities of daily living. Limitations in a fundamental daily life activity such as walking would have an impact on quality of life in this population [50]. With respect to age and type of MS, the published data suggest that the clinical phenotype and course of MS are age dependent; thus, disability accumulation might be an effect of age [51,52]. Lastly, our results showed significant differences in the web-based form score for the number of comorbidities, which could be because comorbidities can negatively impact patients with MS, including the acceleration of disability progression [53]. All these associations are in line with the study reported by Nicholl et al. [17] for the BI validated in patients with MS and with the validity of the translated version of the BI into various languages [19,20,54] in other conditions. Thereby, the construct validity of the Spanish version is corroborated.

Test-retest analyses demonstrated that the web-based form of the BI was stable for detecting and observing functional disability changes in patients over time in clinical settings. The agreement rates of the web-based form of individual items were ≥92%, which was a satisfactory level, and that of the total scores was confirmed to be high (ICC = 0.97). These results are similar to those of previous test–retest reliability studies performed in stroke survivors [55,56]. We are aware that there are many differences between these two conditions and therefore they cannot be compared. However, the patients of these two studies were all more than six months post-stroke onset and therefore it might be anticipated that their functional status would be stable over the short time between assessments similar to our clinically stable patients with MS. To our knowledge, no previous studies have established the SEM and MDC of the BI in patients with MS, so we could not compare our findings. Despite not being comparable, in other neurological pathologies, such as in patients after stroke, Yang et al. [55] reported a similar MDC to that obtained in our study (MDC = 12.09 vs. 16.2). The results of our study help us determine whether there have been real and clinically relevant changes in functional disability (as measured by the BI) in patients with MS. It is therefore possible to determine whether patients have limitations in performing daily activities and to identify the effectiveness of therapies aimed at alleviating or improving these aspects.

### 4.1. Clinical Implications

The web-based form of the BI scale could be a suitable alternative in certain circumstances to remotely examine and follow the disability of patients with MS, preventing unnecessary trips to outpatient clinics and/or health centers. With the rise of telemedicine, it is essential to have validated tools to assess the evolution of this pathology. The MDC established in this study will allow us to determine whether the changes produced in the disability of patients with MS are real and could have clinical repercussions. Thus, the web-based form of the BI allows telematic assessment for detecting functional disability changes in patients over time and can determine the efficacy of various interventions. In addition, the use of electronic data capture for health assessment offers data management capabilities (data checking and analysis, reducing the number of missing values or incomplete data or out-of-range values or typing or copying errors) [57].

### 4.2. Limitations

As a limitation, most of our sample presented a moderate disability status and had a relapsing-remitting clinical subtype. Thus, we did not cover the full disability and type of MS range that presents in the MS population, preventing the validity of the scale from being evaluated in these groups. However, it can be confidently assumed that the current study population represents the normal variation of the disease in our country [58]. Further studies could be conducted prospectively in a sample with representative proportions of patients in terms of clinical subtypes of MS. On the other hand, the fact that the BI web version and face-to-face interview were administered on a single day could have caused some recall bias. This approach was used to minimize any change that might occur in the patient’s functional disability attributable to the electronic version assessment and the face-to-face interview being conducted on different days. As in other studies [59,60,61], a reasonable amount of time was left between the completion of both versions so that patients could perform other activities and minimize the recall effect. However, we cannot rule out with certainty the absence of such bias.

## 5. Conclusions

The Spanish web-based form of the BI is a valid and reliable tool to assess the degree of functional disability in patients with MS. The Spanish version of the BI can be applied in both the web-based format and the face-to-face interview format, given that the concordance between them was excellent. In a clinical context, total score differences of more than 12 points could be considered real, indicating a deterioration or improvement in the patient’s functionality.

## Figures and Tables

**Table 1:** Sociodemographic and clinical characteristics of the participants (N = 143 [84♀, 59♂]).

Outcomes	N (%)	Mean ± SD	Min–Max
**Sociodemographic Characteristics**			
Age (years)		49.77 ± 9.07	25–71
Gender (female)	84 (58.7%)		
Weight (kg)		70.22 ± 14.57	43–110
Height (cm)		169.10 ± 9.19	148–188
Level of education			
School graduate	20 (14.0%)		
Intermediate training cycle	38 (26.6%)		
Higher level training cycle	23 (16.1%)		
University degree	62 (43.4%)		
Work status			
Working	54 (37.8%)		
Not working	89 (62.2%)		
**Clinical Characteristics**			
Type of Multiple Sclerosis			
Relapsing-remitting	74 (51.7%)		
Primary progressive	33 (23.1%)		
Secondary progressive	36 (25.2%)		
Disability status (EDSS)		5.34 ± 2.04	0–9
Mild	30 (21.0%)		
Moderate	78 (54.5%)		
Severe	35 (24.5%)		
Health-related quality of Life (EQ-5D-5L)			
Index score (0–1)		0.519 ± 0.341	–0.400–1.000
Visual Analogue Scale (0–100)		57.66 ± 18.95	10–100
Comorbidities			
0	59 (41.3%)		
1–2	71 (49.6%)		
≥3	13 (9.1%)		
Urinary disturbance (yes)	92 (64.3%)		

EDSS, Expanded Disability Status Scale; EQ 5D-5L, European Quality of Life-5 Dimensions; SD, Standard Deviation; ♀, Female; ♂, Male.

**Table 2:** Agreement between the web-based form and the face-to-face interview of the Barthel index for each item and for the total score of the categorical ranking.

Agreement for Each Item							
Web-Based Form		Face-to-Face Interview				Kappa (95%CI)	% Agreement
		0 Points	5 Points	10 Points	15 Points		
Bathing						0.92 (0.85–0.99)	96%
	0 points	44	3				
	5 points	2	94				
Stairs						0.86 (0.79–0.92)	88%
	0 points	56	4	1			
	5 points	5	26	3			
	10 points	1	3	44			
Dressing						0.92 (0.87–0.97)	94%
	0 points	25	0	0			
	5 points	2	28	5			
	10 points	0	2	81			
Mobility						0.95 (0.91–1.00)	96%
	0 points	13	0	0	0		
	5 points	1	14	0	0		
	10 points	0	0	5	1		
	15 points	0	1	2	106		
Transfer						0.99 (0.96–1.00)	99%
	0 points	3	0	0	0		
	5 points	0	14	0	0		
	10 points	0	1	2	0		
	15 points	0	0	0	123		
Feeding						0.88 (0.80–0.96)	94%
	0 points	8	0	0			
	5 points	0	47	6			
	10 points	1	2	79			
Toilet use						0.89 (0.81–0.97)	94%
	0 points	16	1	0			
	5 points	1	5	3			
	10 points	0	3	114			
Grooming						0.91 (0.78–1.00)	99%
	0 points	11	1				
	5 points	1	130				
Bladder						0.91 (0.84–0.97)	94%
	0 points	52	0	2			
	5 points	3	5	1			
	10 points	2	1	77			
Bowels						0.92 (0.86–0.98)	96%
	0 points	10	0	0			
	5 points	1	29	3			
	10 points	0	2	98			
**Agreement for Categorical Ranking of the Total Score**							
**Web-Based Form**	**Face-to-Face Interview**					**Kappa (95%CI)**	**% Agreement**
	**Physically** **Independent**	**Mildly** **Disabled**	**Moderately Disabled**	**Severely Disabled**	**Very Severely Disabled**		
						0.87 (0.81—0.93)	87%
Physically Independent	27	3	3	0	0		
Mildly disabled	3	7	5	0	0		
Moderately disabled	1	2	59	2	0		
Severely disabled	0	0	0	22	0		
Very severely disabled	0	0	0	0	9		

**Table 3:** Spearman correlations of the web-based form of the Barthel index total score with patients’ health-related quality of life, as well as with their disability status, age, and clinical characteristics.

Outcomes	Barthel Index Web-Based Form
Health-related quality of life (EQ 5D-5L)	
Index score	0.687 **
Visual Analogue Scale	0.429 **
Disability Status	
Expanded Disability Status Scale (EDSS)	0.769 **
Age (years)	–0.391 **
Gender	0.148
Type of Multiple Sclerosis	–0.415 **
Urinary disturbance (yes/no)	0.426 **
Comorbidities (n)	–0.247 **

EQ 5D-5L, European Quality of Life-5 Dimensions. ** *p* < 0.01

**Table 4:** Multiple comparisons between participants with different scores in the web-based form of the Barthel index for gender, number of comorbidities, disability status and health-related quality of life.

	Web-Based Form of the Barthel Index Mean ± SD Median (First Quartile–Third Quartile)					
	Total Score (n = 143)	Categorical Ranking of Total Scoring				
		Physically independent (n = 33) ^a^	Mildly disabled (n = 15) ^b^	Moderately disabled (n = 64) ^c^	Severely Disabled (n = 22) ^d^	Very Severely Disabled (n = 9) ^e^
Gender						
Male (n = 59)	72.20 ± 25.33 75 (60–90)	—	—	—	—	—
Female (n = 84)	77.98 ± 25.87 87.5 (70–95)	—	—	—	—	—
Disability status (EDSS)		3.26 ± 1.92 3 (1.75–5.25) ^c,d,e^	4.40 ± 1.67 4.5 (3–6) ^c,d,e^	5.65 ± 1.31 5 (6–6.5) ^a,b,d,e^	6.86 ± 0.73 7 (6–7.12) ^a,b,c,e^	8.61 ± 0.65 9 (8–9) ^a,b,c,d^
Mild MS (n = 30) ^f^	97.00 ± 4.47 100 (95–100) ^g,h^	—	—	—	—	—
Moderate MS (n = 78) ^g^	81.02 ± 16.40 85 (75–95) ^f,h^	—	—	—	—	—
Severe MS (n = 35) ^h^	45.14 ± 26.64 45 (20– 70) ^f,g^	—	—	—	—	—
Comorbidities						
None (n = 60) ^i^	80.33 ± 23.70 90 (70–100) ^j^	—	—	—	—	—
1 or 2 (n = 34)	76.62 ± 27.68 87.5 (70–95)	—	—	—	—	—
2 or more (n = 49) ^j^	69.08 ± 25.83 75 (50–87.5) ^i^	—	—	—	—	—
Health-related quality of life (EQ-5D-5L)						
Index score (n = 143)	—	0.779 ± 0.174 0.799 (0.684–0.867) ^b,c,d,e^	0.629 ± 0.263 0.675 (0.523–0.800) ^a,d,e^	0.560 ± 0.245 0.591 (0.502–0.738) ^a,d,e^	0.183 ± 0.290 0.176 (0.050–0.399) ^a,b,c,e^	−0.090 ± 0.295 −0.228 (−0.289–0.092) ^a,b,c,d^
Visual Analogue Scale (n = 143)	—	69.64 ± 18.33 75 (60–80) ^b,c,d,e^	58.70 ± 15.87 60 (50–75) ^a,e^	57.28 ± 15.73 50 (50–70) ^a,e^	50.25 ± 19.25 50 (40–60) ^a,e^	32.78 ± 15.02 30 (25–40) ^a,b,c,d^

EDSS, Expanded Disability Status Scale; EQ 5D-5L, European Quality of Life-5 Dimensions; MS, Multiple Sclerosis; SD Standard Deviation. Superscript letters (from “a” to “j”) indicate the groups in which statistically significant differences were found according to post-hoc comparisons using Mann-Whitney U test.

**Table 5:** Test-retest reliability for each item and for the total score of the categorical classification of the web-based form of the Barthel index.

Agreement for Each Item							
Web-Based Form		Web-Based Form after 7–10 Days				Kappa (95%CI)	% Agreement
		0 Points	5 Points	10 Points	15 Points		
Bathing						0.82 (0.71–0.93)	93%
	0 points	32	4				
	5 points	5	80				
Stairs						0.89 (0.82–0.96)	92%
	0 points	47	1	1			
	5 points	1	29	2			
	10 points	1	4	35			
Dressing						0.95 (0.91–1.00)	97%
	0 points	18	0	0			
	5 points	0	31	1			
	10 points	0	3	68			
Mobility						0.92 (0.85–0.99)	95%
	0 points	9	0	0	0		
	5 points	1	12	0	0		
	10 points	0	0	5	0		
	15 points	0	2	3	89		
Transfer						0.93 (0.84–1.00)	98%
	0 points	3	0	0	0		
	5 points	0	11	0	0		
	10 points	0	0	2	0		
	15 points	0	2	0	103		
Feeding						0.86 (0.77–0.95)	92%
	0 points	7	0	0			
	5 points	0	37	5			
	10 points	0	5	67			
Toilet use						0.86 (0.76–0.96)	93%
	0 points	11	2	0			
	5 points	0	5	2			
	10 points	0	4	97			
Grooming						0.89 (0.74–1.00)	98%
	0 points	9	1				
	5 points	1	110				
Bladder						0.90 (0.82–0.97)	93%
	0 points	42	1	1			
	5 points	0	5	3			
	10 points	3	0	66			
Bowels						0.90 (0.82–0.97)	94%
	0 points	9	1	0			
	5 points	0	23	1			
	10 points	0	5	82			
**Agreement for Categorical Ranking of the Total Score**							
**Web-Based Form**	**Web-Based Form after 7–10 days**					**Kappa (95%CI)**	**% Agreement**
	**Physically** **Independent**	**Mildly** **Disabled**	**Moderately Disabled**	**Severely Disabled**	**Very Severely Disabled**		
						0.83 (0.75–0.92)	86%
Physically Independent	19	4	3	2	0		
Mildly disabled	2	7	6	0	0		
Moderately disabled	0	0	56	0	0		
Severely disabled	0	0	0	14	0		
Very severely disabled	0	0	0	0	8		

## Data Availability

Not applicable.

## Supplementary Materials

The following supporting information can be downloaded at: https://www.mdpi.com/article/10.3390/ijerph192113965/s1. Table S1. The Spanish web-based form of the Barthel index.
