# Supplementary material for: Development of the Web-Based Spanish Version of the Barthel Index in Patients with Multiple Sclerosis
Source: Int J Environ Res Public Health. 2022 Oct 27;19(21):13965. doi: 10.3390/ijerph192113965 (PMC9657693; doi:10.3390/ijerph192113965)
Supplement: Supplementary file 1 [file ijerph-19-13965-s001.zip › ijerph-1943032-supplementary.pdf]

Table S1. The Spanish web-based form of the Barthel index.

Estas son algunas preguntas sobre tu capacidad de cuidarte a ti mismo. Puede parecer que no se aplican a usted, no obstante, le pedimos por favor que conteste a todos los ítems con la opción más aplicable a su situación personal. Por favor, marque una casilla en cada sección.	Puntuación
1. En el baño o en la ducha, usted…	
☐ Se maneja por sí mismo sin ayuda	5 puntos
☐ Necesita ayuda para entrar y salir	0 puntos
☐ Necesita otro tipo de ayuda (p. ej. enjabonarse, aclararse, mantener el equilibrio, etc)	0 puntos
☐ Nunca se baña o ducha	0 puntos
☐ Necesita que le aseen/laven en la cama	0 puntos
2. ¿Cómo sube las escaleras de su casa?	
☐ Sin ayuda	10 puntos
☐ Con alguien que le lleve su andador, bastón u otra ayuda técnica	5 puntos
☐ Con alguien que le supervise, motive y/o le dé indicaciones verbales	5 puntos
☐ Con alguien que le ayude físicamente	5 puntos
☐ No sube ni baja escaleras	0 puntos
☐ No tiene escaleras	0 puntos
3. ¿Cómo se viste?	
☐ Sin ninguna ayuda	10 puntos
☐ Únicamente con ayuda para abrocharse los botones/corchetes, cordones y/o cremalleras	5 puntos
☐ Con alguien ayudándole la mayor parte del tiempo	0 puntos
4. ¿Cómo camina en espacios interiores?	
☐ Sin ayuda, exceptuando el uso de un andador/bastón o similar (si es que utiliza)	15 puntos
☐ Con la supervisión de una persona (sin contacto/ayuda física)	10 puntos
☐ Con ayuda de otra persona	10 puntos
☐ Con ayuda de más de una persona	0 puntos
☐ No camina en espacios interiores/cerrados	0 puntos
☐ Emplea una silla de ruedas de manera independiente (p. ej. para girar esquinas)	5 puntos
5. ¿Cómo se traslada de la cama a la silla?	
☐ Sin ayuda	15 puntos
☐ Con una ligera ayuda de otra persona	10 puntos
☐ Con mucha ayuda, prestada por una o varias personas	5 puntos
☐ No lo hace, o requiere de una grúa para hacerlo	0 puntos
6. En el supuesto de disponer de comida al alcance de la mano, usted puede comerla…	
☐ Sin ayuda	10 puntos
☐ Si se la han preparado previamente (p. ej. se la han cortado o le han untado la mantequilla)	5 puntos
☐ Si le prestan mucha ayuda (más de la descrita en las otras respuestas; p. ej. llevarle la comida a la boca)	0 puntos
7. ¿Cómo usa el retrete/inodoro? Entendiendo que dicha acción requiere entrar y salir del baño, así como limpiarse y vestirse	
☐ Sin ayuda	10 puntos
☐ Con un poco de ayuda, pero puede realizar parte de la actividad solo	5 puntos
☐ Con mucha ayuda	0 puntos
8. ¿Cómo puede realizar usted las siguientes acciones relacionadas con el aseo personal? Lavarse la cara/manos/dientes, peinarse y/o afeitarse	
☐ Sin ayuda	5 puntos
☐ Con ayuda	0 puntos
9. ¿Tiene incontinencia de orina?	
☐ No, nunca	10 puntos
☐ Menos de una vez a la semana	10 puntos
☐ Menos de una vez al día	5 puntos
☐ Con más frecuencia	0 puntos
☐ Tiene un catéter/sonda administrado por usted mismo	0 puntos
10. ¿Tiene incontinencia fecal?	
☐ No, nunca	10 puntos
☐ Excepcionalmente, tiene un accidente por semana o menos	5 puntos
☐ Diariamente, siempre	0 puntos
☐ Necesita que alguien le ponga/suministre un enema para evacuar	0 puntos
